# Increasing the frequency of peripheral component in paired associative stimulation strengthens its efficacy

**DOI:** 10.1038/s41598-019-40474-0

**Published:** 2019-03-07

**Authors:** Aleksandra Tolmacheva, Jyrki P. Mäkelä, Anastasia Shulga

**Affiliations:** 1 BioMag Laboratory, HUS Medical Imaging Center, University of Helsinki and Helsinki University Hospital, P.O. Box 340, FI-00029 Helsinki, Finland; 2 Clinical Neurosciences, Neurology, University of Helsinki and Helsinki University Hospital, P.O. Box 372, FI-00029 Helsinki, Finland

## Abstract

Paired associative stimulation (PAS), a combination of transcranial magnetic stimulation (TMS) with peripheral nerve stimulation (PNS), is emerging as a promising tool for alleviation of motor deficits in neurological disorders. The effectiveness and feasibility of PAS protocols are essential for their use in clinical practice. Plasticity induction by conventional PAS can be variable and unstable. Protocols effective in challenging clinical conditions are needed. We have shown previously that PAS employing 50 Hz PNS enhances motor performance in chronic spinal cord injury patients and induces robust motor-evoked potential (MEP) potentiation in healthy subjects. Here we investigated whether the effectiveness of PAS can be further enhanced. Potentiation of MEPs up to 60 minutes after PAS with PNS frequencies of 25, 50, and 100 Hz was tested in healthy subjects. PAS with 100 Hz PNS was more effective than 50 (*P* = 0.009) and 25 Hz (*P* = 0.016) protocols. Moreover, when administered for 3 days, PAS with 100 Hz led to significant MEP potentiation on the 3^rd^ day (*P* = 0.043) even when the TMS target was selected suboptimally (modelling cases where finding an optimal site for TMS is problematic due to a neurological disease). PAS with 100 Hz PNS is thus effective and feasible for clinical applications.

## Introduction

Paired associative stimulation (PAS) is a noninvasive technique that combines peripheral electrical nerve stimulation (PNS) of the limbs and transcranial magnetic stimulation (TMS) of the motor cortex^1,2^. PAS was initially designed for the investigation of human synaptic plasticity and was later applied to clinical studies to develop new rehabilitation tools for neurological disorders, such as incomplete spinal cord injury and stroke^3^. Spinal PAS aims to deliver TMS and PNS so that centrally and peripherally induced volleys collide at the level of the spinal cord^4–6^. The repetitive coincidence is thought to induce a long-term potentiation (LTP^7^)-like effect in the spinal cord.

The current recommendation for a conventional PAS protocol includes a single TMS pulse at the optimum cortical stimulation site (hotspot) of the stimulated muscle at an intensity producing a MEP of 1 mV in a small hand muscle^3^. PNS is delivered either as single pulses^1^ or in 10-Hz trains^8^. Conventional PAS may employ fixed interstimulus intervals (ISIs) between TMS and PNS^2^, or if individually adjusted, requires exact ISI determination (e.g., by MEP and C-root latencies) to ensure correct timing of the stimuli^9^. The outcome of conventional PAS depends strongly on various conditions, such as time of day, subject characteristics, and activities before PAS^3^. One of the problems in the field is the low predictability of the presence, type, and magnitude of the PAS response^3^.

Optimizing PAS can be challenging even in healthy subjects. This is even more so in neurological patients, as the central nervous system (CNS) pathology may modify the signal conduction in the targeted neural pathways. For example, defining the appropriate ISI between TMS and PNS and mapping of the motor cortex to identify the TMS target can be problematic after spinal cord injury due to abnormal nerve conductivity and altered physiology of the spinal cord and the motor cortex^10^. Additionally, muscle spasticity, associated with many neurological diseases, results in a poor signal-to-noise ratio of electromyography (EMG). It is therefore challenging to detect MEPs (usually with an abnormal latency and shape) from spasticity-contaminated EMG^11^. To overcome these issues, we previously employed a PAS protocol consisting of a high-frequency PNS train and high-intensity TMS. This modification provided a wider range of ISIs for effective PAS^11–13^ and is beneficial in chronic SCI patients when applied as a long-term treatment. This leads to a long-lasting increase in the motor scores of individual muscles and produces functional benefits for the patients^11,12^.

Here we report further modifications of PAS that enhance its efficacy. We have previously hypothesized that high-frequency PNS contributes to the effectiveness of the PAS protocol^13^ as it enables multiple collisions of orthodromic and antidromic action potential chains. Here we further tested this hypothesis by comparing previously utilized 50 Hz PNS^11–13^ to higher (100 Hz) and lower (25 Hz) PNS frequencies to detect settings that elicit the strongest MEP potentiation. We also tested our most effective protocol in the condition mimicking the situation in patients whose optimal TMS target in the cortex cannot be precisely identified.

## Methods

### Subjects

We recruited healthy subjects without contraindications to TMS. The study was approved by the medical ethical committee of the Helsinki University Hospital (HUS/1280/2016). All experiments were performed in accordance with relevant guidelines and regulations. All subjects signed an informed consent form. The subjects were asked to avoid caffeine intake for 6 hours prior to the measurements and not to engage in extremely intensive physical activity for 1 day prior to the measurements.

### Transcranial magnetic stimulation

We administered TMS with a figure-8-coil with eXimia magnetic stimulator (Nexstim Ltd., Helsinki, Finland). The accuracy of the stimulus delivery was ensured with an incorporated navigation system based on magnetic resonance imaging (MRI). Navigated TMS (nTMS) displays a dynamic estimate of the stimulus-induced electric field on the patient’s individual 3-D brain MRI reconstruction, enabling selection and precisely repeatable stimulation of localized targets^14–16^. Additionally, we marked the head tracker of the navigation system placed on the subject’s head with a felt pen to prevent shifting during the experiment. To map cortical representations of the target muscle, the TMS coil was placed over the primary motor cortex (M1). Suprathreshold stimuli were applied throughout the leg or hand M1. The elicited MEPs were recorded with an EMG device integrated to eXimia magnetic stimulator (band-pass filter 10–500 Hz, sampling rate 3 kHz). The site in the M1 where TMS produced the largest and most consistent MEPs (peak-to-peak amplitude) and visible movement in the target muscle was identified as the muscle hotspot. Resting motor threshold (RMT) of the hotspot was identified as the lowest TMS intensity that elicited MEPs of at least 50 µV (peak-to-peak amplitude) in at least 5/10 attempts. MEP latency was defined as the onset of the response where signal deviated above the baseline noise level and calculated as a mean of latencies of 10 MEPs elicited by TMS with 120% RMT intensity. This latency was used for calculation of the ISI between PNS and TMS^17^.

### PNS and F-responses

We used a Dantec Keypoint device (Natus Medical Inc., Pleasanton, CA) for peripheral stimulation. For tibial nerve stimulation (for PAS and F-response recording), two surface electrodes (Neuroline 720, AMBU A/S, Ballerup, Denmark) were placed on the medial surface of the right ankle joint behind the malleolus. For median nerve stimulation, two surface electrodes were placed on the middle of the wrist. Eight subjects applied a 2.5% lidocain/prilocain cream (Emla)^18^ to the stimulated area before the experiment to prevent unpleasant sensations during PNS^13^. To record F-responses, the recording electrode was placed over the bulk of the right abductor hallucis (AH) or abductor pollicis brevis (APB). The reference electrode was placed on the proximal/medial surface of the right big toe or proximal/lateral surface of the thumb. We defined the shortest latency out of 10 F-responses evoked with a single 0.2-ms pulse at suprathreshold intensity. This latency was used for calculation of ISI between PNS and TMS^17^. We defined a minimum PNS intensity that reliably elicited F-responses with a single 1-ms pulse and used this individual intensity for PNS in the PAS protocol (for a detailed method description see^13,17^).

### PAS

**Figure 1:**
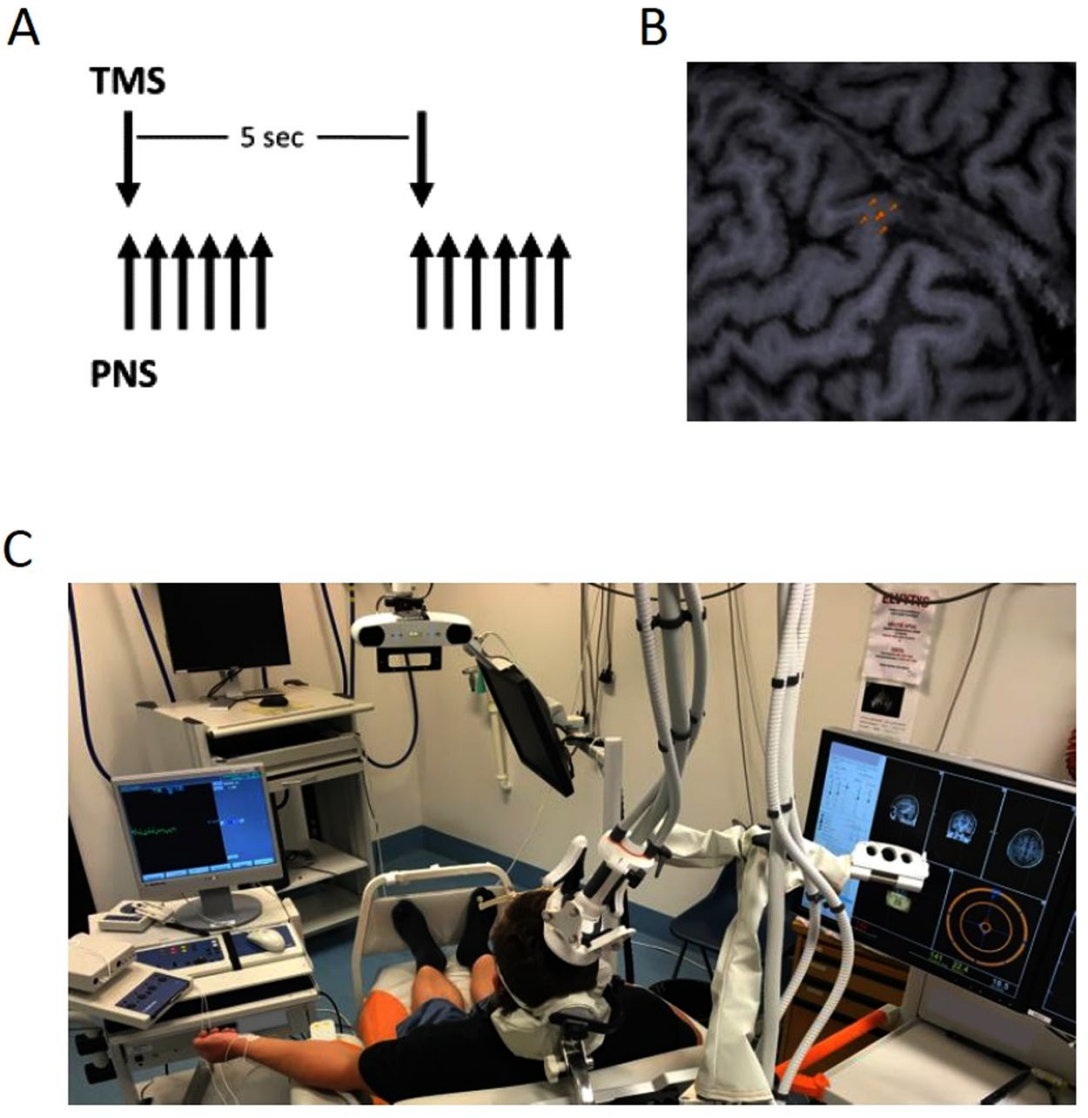
Paired associative stimulation protocol. (**A**) The single TMS pulse, delivered once every 5 s, is synchronized at the spinal level with the first pulse of the PNS train, which consists of 6 pulses delivered at 25, 50, or 100 Hz. (**B**) Five selected sites in the M1 in Experiment 2 in a representative subject. The hotspot of the target muscle (AH) is in the center; four surrounding sites placed equidistant from the central site form a square and represent suboptimal sites of the target muscle. (**C**) PAS setup.

PAS consisted of TMS and PNS paired every 5 s (0,2 Hz, Fig. 1). ISI was calculated for each subject using a formula (ISI = F-response latency–MEP latency) as previously described^17^. The calculated ISI is thought to provide synchronous arrivals of the first descending activity induced by TMS and first ascending activity induced by PNS at the spinal cord level (Fig. 1A). TMS and PNS were triggered with Presentation software (Neurobehavioral System Inc., Albany, NY). Single-pulse TMS was applied at 100% of maximum stimulator output (MSO)^13^. PNS was administered as a train consisting of six 1-ms square wave pulses at 25 Hz, 50 Hz, or 100 Hz in Experiment 1 (see below) and at 100 Hz in Experiment 2 (based on the results of Experiment 1). One PAS session consisted of 240 paired pulses (20 min). Consistent with the protocol used in clinical studies, all subjects were asked to imagine plantar flexion of the stimulated foot during tibial nerve stimulation and abduction of the stimulated thumb during median nerve stimulation^11,12^. The subjects were not evaluated for motor imagery capacity.

### Experimental Design

The aim of Experiment 1 was to study the impact of PNS frequency on the effectiveness of the PAS protocol. For Experiment 1, we recruited 10 healthy subjects (5 males, age range 23–40 years, mean age 32 years). Each subject underwent three PAS sessions with at least a 1-week interval in between in a random order. We tested three different PAS protocols. PNS was applied to the right tibial nerve with frequencies of 25, 50, and 100 Hz (PAS/25, PAS/50, PAS/100); TMS was delivered to the motor cortex hotspot of the right AH muscle. The subjects were not informed about the type of stimulation protocol or the purpose of the experiment. We recorded 30 MEPs from right AH before, immediately after (0 min), 30 min, and 60 min after the stimulation. We also applied PNS only at 100 Hz as a control PNS experiment in 5 subjects. Additionally, we applied PAS/100 to the upper extremity in 5 subjects. PNS was delivered to the right median nerve and TMS to the motor cortex hotspot of the right APB.

The aim of Experiment 2 was to test the most effective protocol as determined in Experiment 1 under conditions where the target for TMS is selected suboptimally. For Experiment 2, we enrolled 5 healthy subjects (3 males, age range 25–61 years, mean age 38 years). We determined the motor cortex hotspot for the right AH. We used a grid function in Nexstim NBS software to select four additional sites equidistant from the hotspot. We placed the hotspot in the center of a grid and selected four surrounding sites in the motor cortex from the grid at 4 to 6 mm from the hotspot (Fig. 1B). TMS delivered to all five sites produced MEPs in the AH. We measured 30 MEPs from each site with a TMS intensity of 120% of the resting motor threshold (RMT) of the hotspot. In this experiment we attempted to mimic the clinical situation where the most optimal stimulation site cannot be determined. Therefore, we detected a triangle of three adjacent sites producing the weakest average MEP at 120% RMT (calculated from 90 MEPs recorded from the 3 sites) and containing the hotspot for further measurements. The site producing the smallest MEPs out of these three sites was selected as the target for sequential PAS. We administered PAS/100 to this weakest site on the 1^st^, 2^nd^, and 3^rd^ day of the experiment and measured MEPs before and immediately after the stimulation. In addition, we measured MEPs on the 8^th^ day after the first PAS session. The subjects were not informed which site was targeted each time.

In both experiments, we evaluated the outcome with MEPs elicited by TMS at 120% RMT of the hotspot. 30 MEPs were recorded with a 3.3-s interval between the TMS pulses. We visually examined EMG in a 200-ms time window before TMS for spike activity (any increase of EMG amplitude exceeding EMG baseline) and discarded EMG-contaminated MEPs to exclude effects of muscle preactivation from the averaged MEPs. In both experiments, MEP potentiation was defined as the percent ratio of an average of post-PAS to pre-PAS MEPs.

### Statistical Analysis

IBM SPSS Statistics 24 software was used for all analyses. In the experiment with n = 10 subjects, we used parametric tests; the data were assessed for normality with Kolmogorov-Smirnov test (p = 0.2). In experiments with n = 5 subjects, we used non-parametric tests. Data are presented as mean ± standard error mean (s.e.m) for all data; in addition, median and 1^st^ and 3^rd^ quartiles (Q1, Q3) are reported for nonparametric data. For multiple comparisons, we performed ANOVA with repeated measures (with frequency as within-subject factor) and Bonferroni post-hoc analysis. We used Wilcoxon signed-rank tests and Friedman test for multiple comparisons in Experiment 2.

## Results

All subjects tolerated the stimulation well. Some of the subjects reported PNS as slightly unpleasant. No unpleasant sensations due to the use of TMS at 100% SO were reported.

**Figure 2:**
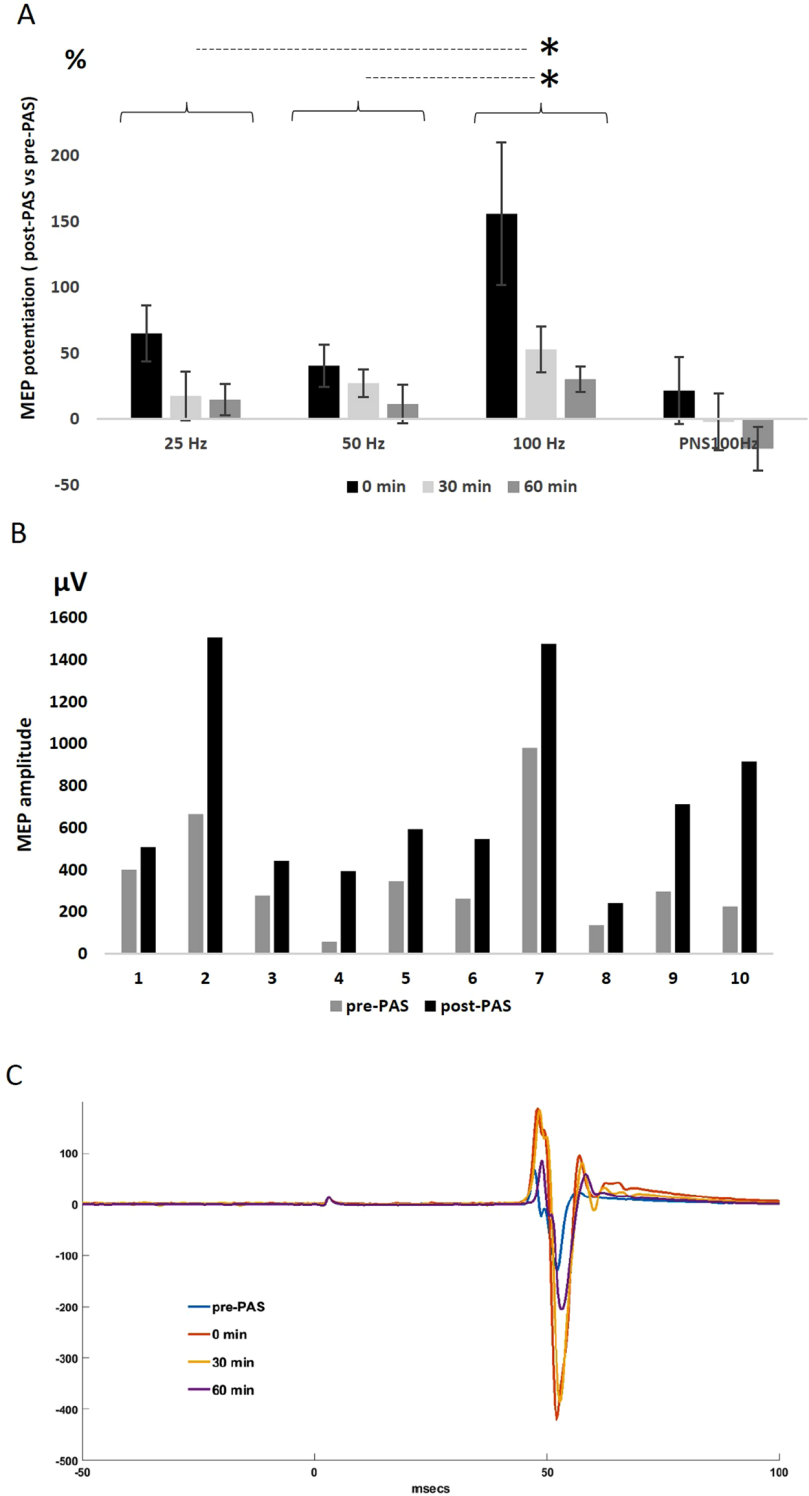
PAS/100 Hz is the most effective in potentiating MEPs. (**A**) MEP potentiation (post-PAS normalized to pre-PAS minus 100%) induced by protocols utilizing PNS of 25, 50, and 100 Hz and control PNS of 100 Hz measured immediately after, 30 min, and 60 min after the stimulation. Data are presented as mean ± s.e.m. (n = 10 subjects). (**B**) MEP potentiation induced by PAS/100 Hz in individual subjects in Experiment 1. MEPs were measured before and after PAS. MEP potentiation was induced in all 10 subjects. (**C**) The average of 30 MEPs in a representative subject before and after PAS/100.

Experiment 1 was conducted to compare the effectiveness of PAS with three different PNS frequencies (Fig. 2A). In an ANOVA with repeated measures with a Greenhouse-Geisser correction for multiple comparison of data from all time points, the mean scores for MEP potentiation were significantly different (F(1.076, 31.210) = 9.488, *P* = 0.004, MEP potentiation for each frequency and time point is shown in Fig. 2A). Pairwise comparisons by Bonferroni post-hoc analysis revealed significant differences between the PAS/100 and PAS/50 protocol (p = 0.009; 158 ± 25% 100 Hz vs 50 Hz at all time points) and the PAS/100 and PAS/25 protocol (*P* = 0.016; 151 ± 17% 100 Hz vs 25 Hz at all time points); no significant difference was seen between PAS/25 and PAS/50. PNS (100 Hz) only did not induce MEP potentiation and there was a trend towards MEP amplitude depression at 60 min (Fig. 2A)^13^. PAS/100 induced MEP potentiation immediately after PAS in 10/10 subjects (Fig. 2B) and at 60 min in 9/10 subjects. Figure 2C shows the averaged MEPs in a representative subject before and up to 60 min after PAS/100.

**Figure 3:**
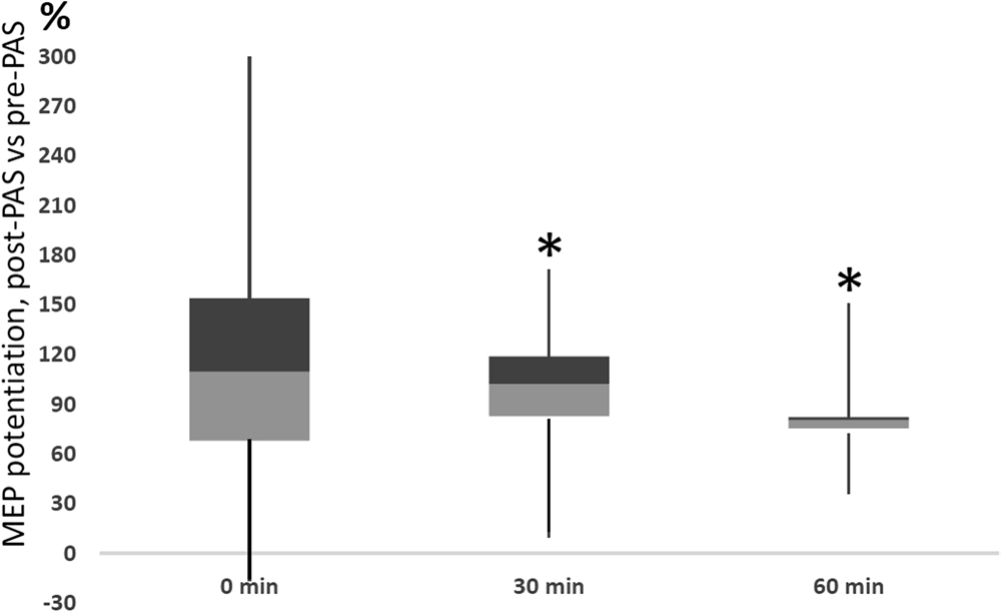
PAS/100 Hz induces MEP potentiation in the upper limb. MEP potentiation (post-PAS normalized to pre-PAS minus 100%) induced by PAS/100 Hz to right abductor pollicis brevis (Experiment 1) measured immediately after, 30 min, and 60 min after the stimulation. Data are presented as box plots showing median and 1^st^ and 3^rd^ quartiles (n = 5 subjects).

We tested whether PAS/100 is also effective in the upper limb (Fig. 3). The PAS/100 protocol administered to the median nerve/right APB hotspot induced a robust MEP potentiation at 30 min and 60 min (Fig. 3; 197 ± 26%, *P* = 0.043; 185 ± 18%, *P* = 0.043, respectively; Wilcoxon signed-rank test; median and quartile data shown in Fig. 3). There was a trend towards MEP increase at 0 min (221 ± 53%, *P* = 0.08).

**Figure 4:**
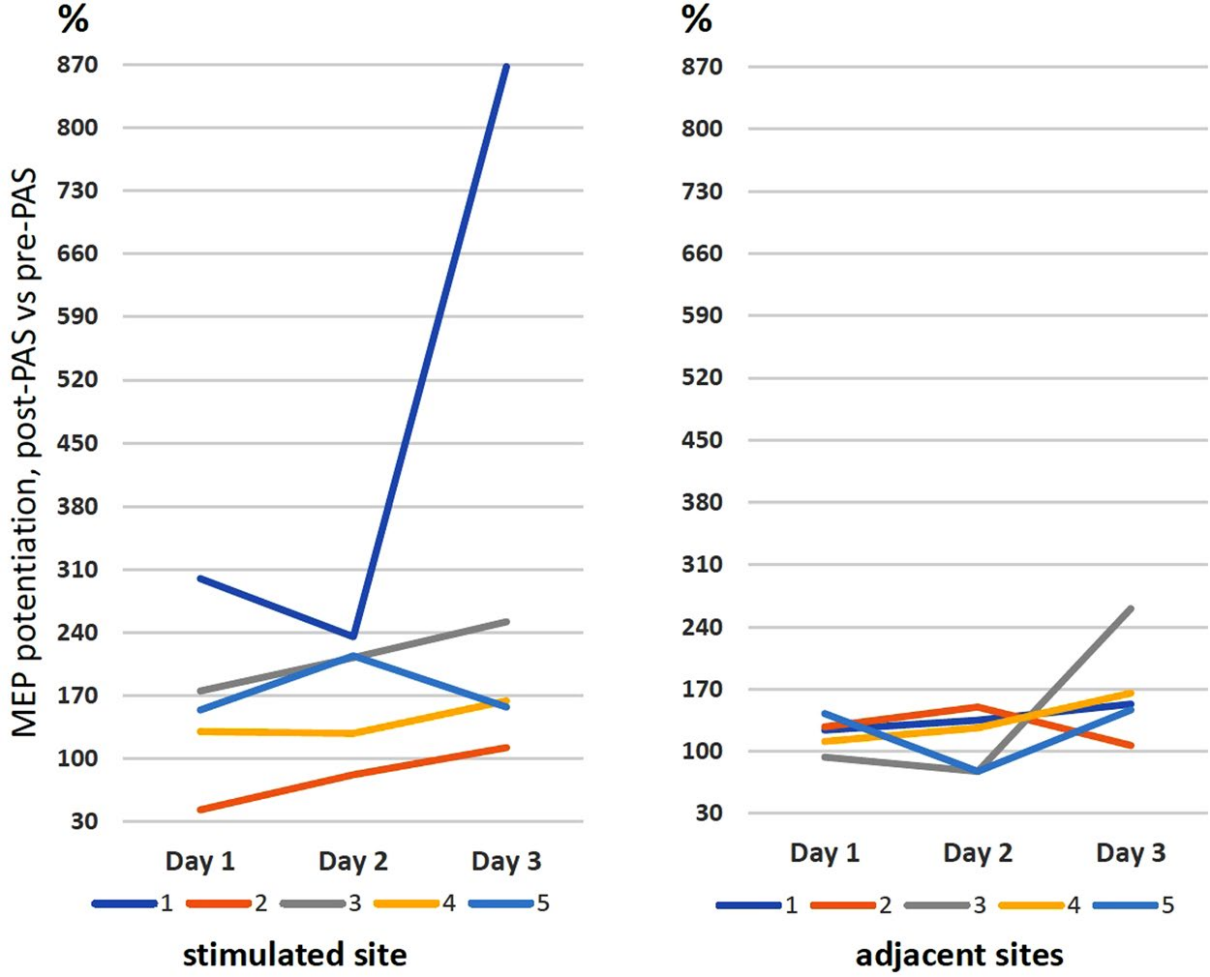
PAS/100 Hz administered for 3 consecutive days induces MEP potentiation even if a suboptimal cortical stimulation site is selected. MEP potentiation (post-PAS vs pre-PAS) is induced by PAS/100 Hz measured from the suboptimal area and adjacent sites in the M1 in all 5 subjects at the 3^rd^ day of stimulation. Individual MEP potentiation data of the 5 subjects (different line colors) is shown.

In Experiment 2 (Fig. 1B and 4), we studied whether PAS/100 would be effective if it was not possible to find the most optimal stimulation site for the TMS. We also studied how PAS/100 affects two adjacent cortical sites to determine if the sites would become potentiated or weakened. We stimulated the weakest site out of 5 sites identified in M1 as producing MEPs (n = 30 MEPs per measurement) from AH. We measured MEP amplitudes from the cortical area (Fig. 1B) consisting of the stimulated site and average MEP amplitude of two adjacent sites (producing stronger MEPs at initial evaluation; n = 2 × 30 = 60 MEPs per measurement). MEP potentiation in both the stimulated and the adjacent sites became significant on the 3^rd^ stimulation day (Fig. 4; 311 ± 141%, median 164%, Q1 157%, Q3 252%, *P* = 0.043 in the stimulated site and 166 ± 26%, median 153%, Q1 147%, Q3 166%, *P* = 0.043 in the average of adjacent sites in Wilcoxon signed-rank test). MEP potentiation was significantly stronger on the 3^rd^ day when compared solely with the 1^st^ day value in the stimulated site (3^rd^ vs 1^st^ day potentiation: 184 ± 38%, median 144%, Q1 126%, Q3 260%, *P* = 0.043; Wilcoxon signed-rank test); this was not observed in the adjacent sites. Individual variation of the potentiation was considerable (Fig. 4). Multiple comparison by Friedman test did not, however, reveal a significant difference in strenght of MEP potentiation between days (*P* = 0.074 for stimulated site and *P = *0.247 for adjacent sites). MEP baseline amplitude in the stimulated site increased significantly from the 1^st^ to the 8^th^ day (8^th^ vs 1^st^ day pre-stimulation amplitude: 209 ± 50%, median 213%, Q1 113%, Q3 231%, *P* = 0.043; Wilcoxon signed-rank test); no significant increase was seen in MEPs from the adjacent sites.

## Discussion

We further improved our modified PAS protocol utilizing high-intensity navigated TMS and high-frequency PNS trains. This type of PAS is not yet well characterized.

Spinal PAS protocols aim to induce spike timing-dependent plasticity (STDP) at the spinal cord level. The STDP model in which synaptic input to dendrites is active just before a somatic action potential is now regarded as highly simplified^3^. Spike timing is not the only requirement for plasticity induction, which depends also on the firing rate, postsynaptic voltage, and synaptic cooperativity^19^. For example, in brain tissue slice experiments, connections exhibited Hebbian STDP only when presynaptic and postsynaptic spikes occurred at moderate firing rates (10–20 Hz); higher firing rates (>30 Hz) induced LTP independent of spike timing^19^.

PAS protocols with 25-, 50-, and 100-Hz PNS trains elicit different firing patterns of postsynaptic membranes and thus provide different conditions for collisions of descending impulse volleys induced by TMS and ascending volleys evoked by the PNS train. The 25-, 50-, and 100-Hz PNS trains gave rise to six distinctive antidromic volleys with interpulse intervals of 40, 20, and 10 ms, respectively. High-intensity TMS can induce one D-wave and four I-waves with an interval of approximately 1.5 ms in between, fitting a time window of no longer than 10 ms^20^. It is plausible that a TMS pulse administered at 100% SO activates several neural populations of variable conductivity^21^. This occurs not only in the stimulated site but also in the neighboring cortex, thus inducing neuronal firing in a slightly different time frame. In neurological patients, this temporal dispersion of responses of different neural populations can be even wider. Increasing the frequency of PNS increases the probability for coincidence of ascending and descending volleys within the effective time window for inducing a LTP-like effect. It was thus plausible that increasing the frequency of stimulation would increase the effectiveness of the protocol, whereas decreasing it would result in a less robust potentiation of MEPs. In line with this proposal, the results of the present study showed that PAS/100 was most efficient of the tested protocols. Consistent with the present data, we have previously shown that PNS (50 Hz) alone has no effect on MEPs; TMS alone also does not lead to MEP potentiation^13^.

In experiment 2, we modeled the situation where detecting the hotspot of the target muscle TMS in the motor cortex is problematic due to neurological disease and, consequently, TMS is delivered to a suboptimal site. We applied PAS with TMS administered to the weakest site (out of the 5 tested sites) of the target muscle in the motor cortex for 3 days. In the study of McKay *et al*.^8^, a conventional PAS protocol was administered for 3 days to the hotspot of the muscle. This resulted in increased excitability and expansion of motor cortex representation of the target muscle for 2 days after the last stimulation session. We observed a significant increase in MEP baseline on the 5^th^ day after the last stimulation session despite selection of the suboptimal site. Two possible explanations could account for this phenomenon. First, it is possible that the motor cortex undergoes plastic reorganization reacting to PAS administered to the suboptimal site. Second, it is possible that our findings represent the result of the weaker activation of the whole motor representation area of the muscle by the wide electric field induced by TMS administered at 100% SO, even when the stimulation is not targeting the hotspot. There was no significant difference between extent of MEP potentiation in stimulated vs adjacent sites.

The limitation of Experiment 2 is the small sample size (n = 5). Studies with a larger number of subjects are needed to unravel the exact mechanisms of the effects observed in this experiment. Here, the main goal of this experiment was to show that the protocol is effective in challenging clinical conditions. Importantly, although low-frequency TMS without PNS is thought to have an inhibitory effect, MEPs elicited by TMS in adjacent sites did not decrease in amplitude. The appearance of significant MEP potentiation on the 3^rd^ day even in a small sample size indicates that the observed effect is robust and might be clinically meaningful.

The motor imagery capacity of the subjects was not assessed. This might have added additional variability to the results. We have previously shown that PAS protocol with high-frequency PNS does not require motor imagery to induce MEP potentiation in healthy subjects^13^. However, this might not be so in neurological patients having high resting motor threshold and requiring motor imagery to lower it. Since the primary purpose of the present experiments and of our previous works^13,17^ conducted in healthy subjects is to optimize the protocol for clinical use in patients, we utilized motor imagery in order to mimic the clinical experiments^11,12^ as precisely as possible.

## Conclusions

We demonstrate the advantage of the PAS/100 protocol compared with the tested PAS/25 and PAS/50 protocols. The efficacy of long-term PAS/50 in incomplete SCI patients has been reported^11,12^. PAS/100 applied as a long-term intervention might be even more beneficial after SCI. We suggest that the PAS protocol with high-intensity TMS and high-frequency PNS components presented here is feasible for clinical use since it is effective even when precise motor cortex mapping and determination of ISI^13^ are not possible due to CNS and muscle abnormalities in neurological patients. Further research conducted on SCI and other neurological patients with application of this PAS protocol is justified.

## Data Availability

The datasets generated during and/or analyzed during the current study are available from the corresponding author on request.
